# Supplementary material for: A web-database of mammalian morphology and a reanalysis of placental phylogeny
Source: BMC Evol Biol. 2007 Jul 3;7:108. doi: 10.1186/1471-2148-7-108 (PMC1941728; doi:10.1186/1471-2148-7-108)
Supplement: Additional File 1 — Website data: The 196 characters first described in [17], along with the supplementary data indicated below, have been joined into a number of HTML files, all of which are linked to the "morphsite_bmc07.html" index page. The embedded links to the supporting files are functional, independent of PC or platform, provided that the directory structure remains intact (i.e., all files remain in a single folder, no subdirectories). Nexus files: Datafiles containing the morphological, indel, and aligned DNA sequence data, linked from the "morphsite_bmc07.html" index page. Table 1. Changes to the multiple sequence alignment of [9]. Table 2. Qualitative character-state corrections to the morphology matrix of [17]. Table 3. Summary of discrepancies between Appendices 1 and 2 of [17]. [file 1471-2148-7-108-S1.zip › asher-BMC07-website/postcran.html]

asher-characterBank


|  |  |  |
| --- | --- | --- |
| Postcranium |  | This work is licensed under a   Creative Commons License. |
| Axial skeleton  122 cervical centra length ;  0 > 2x thoracic 1 similar thoracic 2 anteroposteriorly compressed,   123 C2 odontoid  ;  0 present 1 reduced,   124 C2 odontoid shape  ;  0 broad 1 narrow-pointed,   125 C3-7 spinous processes  ;  0 present 1 reduced,   126 ventrum of sternum ;  0 flat 1 keeled,   127 sternal ribs  ;  0 unossified 1 ossified,   128 clavicle ;  0 elongate 1 stout 2 absent,   129 dorsum proximal ribs ;  0 round 1 flattened,   130 intervertebral articulations ;  0 nomarthrous 1 xenarthrous,   131 T5-T9 spinous processes  ;  0 present 1 reduced, | Pectoral girdle and forelimb  132 scapular shape ;  0 craniocaudally long 1 mediolaterall long 2 mediolaterally long and narrow,   133 scapular coracoid  ;  0 reduced 1 ventral glenoid 2 anterior glenoid,   134 radius-humerus ratio  ;  0 similar 1 radius longer,   135 humeral head ;  0 below tuberosity 1 even tuberosity 2 superior tuberosity,   136 humeral supinator ridge ;  0 present 1 reduced,   137 entepicond foramen ;  0 present 1 absent,   138 olecranon fossa ;  0 solid 1 fenestrated,   139 medial epicondyle ;  0 reduced 1 present 2 elongate,   140 distal humerus;  0 cylindrical 1 V-shaped troch,   141 proximal radius  ;  0 capitulum 1 trochlea and capitulum,   142 proximal ulna  ;  0 single surface 1 notched for radius,   143 ulnar olec ;  0 olec-humeral artic 1 reduced olecranon,   144 distal ulna;  0 no carpal articulation 1 carpal articulation,   145 ulna-radius shaft  ;  0 separate 1 fused,   146 distal radius-ulna  ;  0 separate 1 fused,   147 metacarpal length  ;  0 much shorter forearm 1 >=33% forearm,   148 carpal arrangement ;  0 alternating 1 no lunar-unciform articulation   149 scaphoid-lunate ;  0 separate 1 fused,   150 3rd phalanx 2nd digit ;  0 ossified 1 unossified,   151 terminal phalanx shape  ;  0 ML narrow 1 ML broad,   152 phalanx number  ;  0 <= three 1 > three, | Pelvic girdle 153 sacral vertebrae ;  0 <= four 1 > four,   154 pubic symphysis ;  0 broad 1 reduced,   155 ilium  ;  0 narrow 1 dorsal process,   156 ischial notch ;  0 open 1 closed,   157 sacral-ilium articulation   ;  0 present 1 absent,   158 anterior process pubis  ;  0 absent 1 present,   159 epipubic bones ;  0 present 1 absent,   160 caudal vertebrae ;  0 elongate 1 reduced, |
| Hindlimb  161 femoral fovea capitis ;  0 central 1 marginal 2 indistinct,   162 femoral neck  ;  0 present head medial shaft 1 absent head above shaft,   163 third femoral trochanter;  0 proximal 1 midshaft 2 reduced,   164 medial trochanteric ridge  ;  0 even 1 enlarged,   165 distal tibia-fibula ;  0 unfused 1 fused,   166 fibular ossification ;  0 ossified 1 incomplete,   167 fibula-calcaneus ;  0 articulation 1 facet small-absent,   168 peroneal process ;  0 blunt 1 large,   169 dorsum astragalus  ;  0 flat 1 trochlea,   170 Sustentacular facet ;  0 separate 1 contact distal astragalus,   171 astragalar cotylar fossa;  0 abs 1 pres,   172 astragalar posteromedial process  ;  0 small or abs 1 prominent,   173 astragalar navicular facet  ;  0 convex 1 saddle 2 trochlea 3 concave,   174 calcaneal facet of astragalus ;  0 plantar 1 lateral,   175 astragalar cuboid contact ;  0 reduced contact 1 contact,   176 proximal cuboid  ;  0 flat 1 stepped,   177 calcar ;  0 absent 1 uropatagial spur 2 calcaneal tuber,  178 pedal digital ray 1  ;  0 weight bearing 1 reduced,   179 pedal digital ray 2  ;  0 weight bearing 1 reduced,   180 pedal digital ray 3  ;  0 weight bearing 1 reduced,   181 pedal digital ray 4  ;  0 weight bearing 1 reduced,   182 pedal digital ray 5  ;  0 weight bearing 1 reduced,   183 1st metatarsal  ;  0 as other digits 1 opposable,   184 metatarsal length  ;  0 shorter tibia 1 approach 50% tibia,   185 hallux proximal phalanx ;  0 as other digits 1 elongate |  |  |
